# Pharmacologic Inhibition of the TGF-β Type I Receptor Kinase Has Anabolic and Anti-Catabolic Effects on Bone

**DOI:** 10.1371/journal.pone.0005275

**Published:** 2009-04-16

**Authors:** Khalid S. Mohammad, Carol G. Chen, Guive Balooch, Elizabeth Stebbins, C. Ryan McKenna, Holly Davis, Maria Niewolna, Xiang Hong Peng, Daniel H. N. Nguyen, Sophi S. Ionova-Martin, John W. Bracey, William R. Hogue, Darren H. Wong, Robert O. Ritchie, Larry J. Suva, Rik Derynck, Theresa A. Guise, Tamara Alliston

**Affiliations:** 1 Department of Internal Medicine, Division of Endocrinology, University of Virginia, Charlottesville, Virginia, United States of America; 2 Graduate Program in Oral and Craniofacial Sciences, University of California San Francisco, San Francisco, California, United States of America; 3 Department of Orthopaedic Surgery, University of California San Francisco, San Francisco, California, United States of America; 4 Department of Materials Science and Engineering, University of California, Berkeley and Materials Science Division, Lawrence Berkeley National Laboratories, Berkeley, California, United States of America; 5 Scios, Inc, Fremont, California, United States of America; 6 Department of Orthopaedic Surgery, Center for Orthopaedic Research, Barton Research Institute, University of Arkansas for Medical Sciences, Little Rock, Arkansas, United States of America; 7 Pfizer RTC, Cambridge, Massachusetts, United States of America; 8 Department of Cell and Tissue Biology, University of California San Francisco, San Francisco, California, United States of America; 9 Institute of Regeneration Medicine, University of California San Francisco, San Francisco, California, United States of America; University of Las Palmas de Gran Canaria, Spain

## Abstract

During development, growth factors and hormones cooperate to establish the unique sizes, shapes and material properties of individual bones. Among these, TGF-β has been shown to developmentally regulate bone mass and bone matrix properties. However, the mechanisms that control postnatal skeletal integrity in a dynamic biological and mechanical environment are distinct from those that regulate bone development. In addition, despite advances in understanding the roles of TGF-β signaling in osteoblasts and osteoclasts, the net effects of altered postnatal TGF-β signaling on bone remain unclear. To examine the role of TGF-β in the maintenance of the postnatal skeleton, we evaluated the effects of pharmacological inhibition of the TGF-β type I receptor (TβRI) kinase on bone mass, architecture and material properties. Inhibition of TβRI function increased bone mass and multiple aspects of bone quality, including trabecular bone architecture and macro-mechanical behavior of vertebral bone. TβRI inhibitors achieved these effects by increasing osteoblast differentiation and bone formation, while reducing osteoclast differentiation and bone resorption. Furthermore, they induced the expression of Runx2 and EphB4, which promote osteoblast differentiation, and ephrinB2, which antagonizes osteoclast differentiation. Through these anabolic and anti-catabolic effects, TβRI inhibitors coordinate changes in multiple bone parameters, including bone mass, architecture, matrix mineral concentration and material properties, that collectively increase bone fracture resistance. Therefore, TβRI inhibitors may be effective in treating conditions of skeletal fragility.

## Introduction

In skeletal development, each bone is formed with a distinctive size, geometry, architecture, and material properties. Among the many growth factors and hormones involved in this process [1]–[3], transforming growth factor-β (TGF-β) is sequestered at high levels in bone matrix and is a critical regulator of osteogenesis [4]. Bone mass is dramatically affected by developmental manipulation of TGF-β signaling in genetically modified mouse models [5]–[9]. In addition to bone mass, TGF-β regulates bone matrix material properties, which impact the ability of bone to resist fracture [10]. However, little is known about the role of TGF-β in the post-natal skeleton, which responds to changes in bone or the environment to retain or improve bone quality, fundamentally defined as the ability to resist bone fracture [11].

The effects of postnatal manipulation of TGF-β signaling on bone mass and quality are difficult to predict based on developmental studies. For example, osteoporosis and bone fragility are observed in mice with increased TGF-β production [6], as well as in those that are deficient in Smad3 [8], [9], a key TGF-β effector. Conversely, other mouse models with reduced TGF-β signaling have increased bone mass and quality [7], [10]. In addition, the roles of TGF-β on the proliferation, differentiation, and apoptosis of cells in both the osteoblast and osteoclast lineages have been extensively studied [4], [12]–[14]. In spite of this wealth of information, the net effect of postnatal TGF-β signaling on bone remains unknown.

The recent development of specific inhibitors of the TGF-β type I receptor (TβRI) kinase that block most if not all TGF-β signaling events [15]–[17] now enables an investigation of this fundamental question. ATP-competitive inhibitors of the TβRI kinase, such as SD-208, can effectively limit TGF-β-mediated lung fibrosis and tumorigenesis in vivo at doses that are too low to exert non-specific effects on other kinases [17]–[20]. Since such inhibitors are in clinical trials for cancer and other disorders, it is crucial to define the effects of TGF-β blockade on the skeleton.

Maintenance of the postnatal skeleton depends on the functional coordination between bone-depositing osteoblasts and bone-resorbing osteoclasts [21]. Both cell populations express and respond to TGF-β, and TGF-β has been suggested to couple osteoblast and osteoclast activity [4]. TGF-β promotes osteoprogenitor proliferation and inhibits terminal osteoblast differentiation, in part by repressing the function of osteogenic transcription factor Runx2 [22]. TGF-β also regulates osteoblast expression of osteoclast regulatory factors m-CSF, RANKL, and OPG [23]–[25], whereas resorbing osteoclasts release and activate matrix-bound latent TGF-β, which feeds back to modulate osteoblast and osteoclast function [26]–[28]. Because the effects of TGF-β on osteoblast and osteoclast function are dynamic, dose-dependent, and specific for each cell type and stage of differentiation [4], [12]–[14], prior studies do not indicate how the cell types present in mature bone will respond to a systemic alteration in TGF-β signaling.

In the current study, we found that the TβRI kinase inhibitor, SD-208, affects osteoblast and osteoclast function to coordinately regulate several bone parameters, resulting in increased bone mass and trabecular bone volume, as well as increased mineral concentration and elastic modulus of bone matrix. This was associated with an increased resistance to vertebral fracture. These results suggest that pharmacologic inhibition of TGF-β signaling may have therapeutic utility in a variety of bone diseases characterized by poor bone quality, low bone mass and a propensity to fracture.

## Results

### Pharmacologic inhibition of the TβRI kinase increases bone mineral density

**Figure 1:**
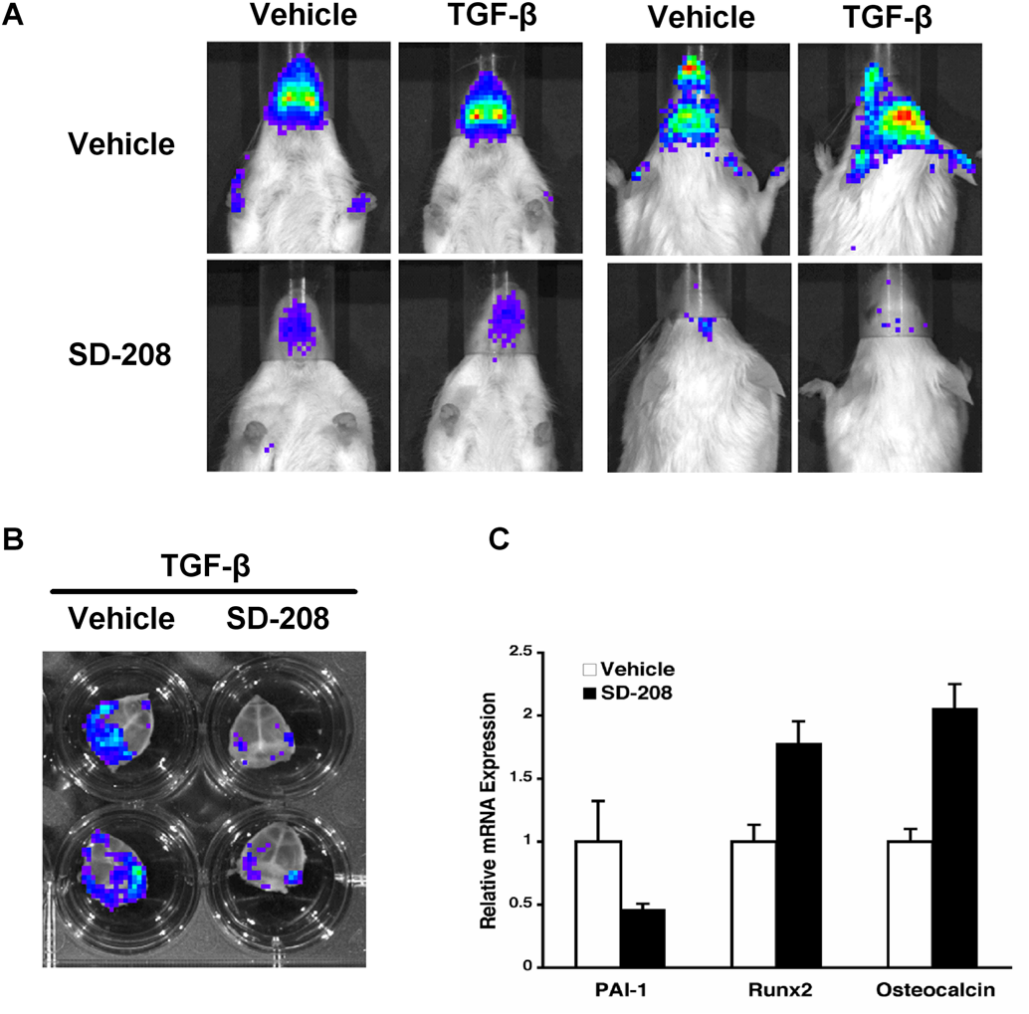
SD-208 inhibition of TGF-β function in vivo. Five hours after TGF-β administration, SBE-Luc mice showed increased bioluminescence on the dorsal and ventral surfaces of the head where relatively little superficial tissue covers skeletal elements (calvarial bone and jaws) (a). Mice pretreated with SD-208 showed less basal and TGF-β-inducible luminescence than vehicle-treated controls (a, lower panels). SD-208 also inhibited reporter activity in SBE-Luc mouse calvarial explants cultured overnight with TGF-β (b). SD-208 treatment of calvarial explants inhibits expression of the TGF-β-inducible gene, PAI-1 [30], but induces expression of Runx2 and osteocalcin, osteoblast marker genes that are targets of TGF-β repression [22].

To determine the effects of pharmacologic inhibition of TGF-β signaling on bone, mice were treated for 6 weeks with either of two doses of SD-208, a small molecule that blocks ATP binding to the type I TGF-β receptor to specifically inhibit its kinase activity [17]. The 20 mg/kg SD-208 dose was chosen to achieve specific inhibition of the TβRI kinase, whereas the 60 mg/kg dose was chosen to achieve a maximal response with minimal inhibition of other pathways [19]. Using mice that express luciferase under the control of a TGF-β-responsive Smad binding element (SBE-Luc mice) [29], we confirmed the ability of SD-208 to inhibit endogenous and exogenously applied TGF-β function in bone in vivo and ex vivo (Fig. 1a, 1b). As expected, the well-established TGF-β-inducible expression of PAI-1 [30] was inhibited by SD-208 in calvarial explants, whereas the expression of reported targets of TGF-β repression, Runx2 and osteocalcin [22], was induced by SD-208 (Fig. 1c).

**Figure 2:**
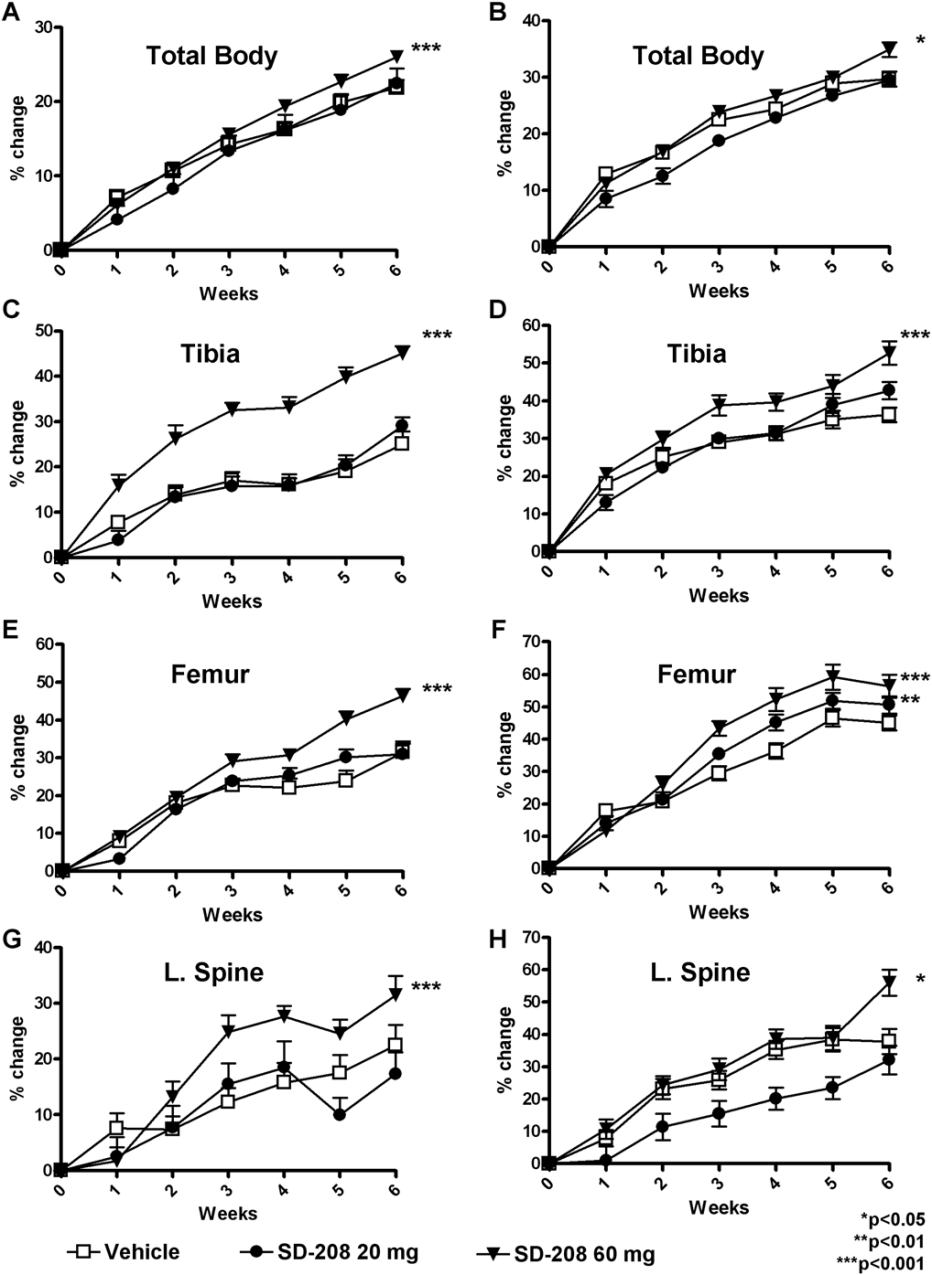
Pharmacologic TβRI inhibition increases BMD. DXA was used to measure BMD longitudinally for male (a, c, e, g) and female mice (b, d, f, h) treated with or without the TβRI inhibitor SD-208 at 20 mg/kg or 60 mg/kg. SD-208 treatment at the 60 mg/kg dose caused an increase in total body (a, b) tibia (c, d), femur (e, f), and lumbar spine (g, h) BMD. SD-208 at the 20 mg/kg dose increased femoral BMD in female mice (f). Data represent mean±SEM (p<0.05, as determined by two-way analysis of variance (ANOVA).

Longitudinal examination of the bone mineral density (BMD) by dual energy X-ray absorptiometry (DXA) showed the normal increase in BMD between 1 and 2.5 months of age. Accordingly, vehicle-treated male and female mice showed an increase of 21.8% and 29.6%, respectively, in whole body BMD after 6 weeks (Fig. 2a, 2b). Although low dose SD-208 did not affect whole body BMD, both male and female mice treated with high dose SD-208, showed significantly greater bone accrual over the same time period, with an additional 4.12% increase in male (p<0.001) and 5.2% increase in female (p<0.001) whole body BMD. The SD-208-induced increase in whole body BMD was comparable to that observed following an 8-week treatment with bisphosphonates, which can increase whole body BMD by 5% [31].

More pronounced effects were apparent in the tibia and femur, where the BMD was already significantly increased within 3 weeks of SD-208 treatment relative to vehicle-treated controls (Fig. 2c–2f). After 6 weeks, SD-208 significantly increased the BMD in male mice by 20% in the tibia (p<0.001), 14.8% in the femur (p<0.001) and 8.9% in the lumbar spine (p<0.01) relative to vehicle-treated mice. SD-208 increased the tibial, femoral and lumbar spine BMD in female mice by 16.3% (p<0.001), 11.4% (p<0.01) and 17.9% (p<0.001), respectively. Dose-dependent increases in BMD were most apparent in the femur (Fig. 2e, 2f). Thus, systemic pharmacologic inhibition of TGF-β signaling increases the BMD.

### Inhibition of the TβRI kinase increases trabecular bone

**Figure 3 pone-0005275-g003:**
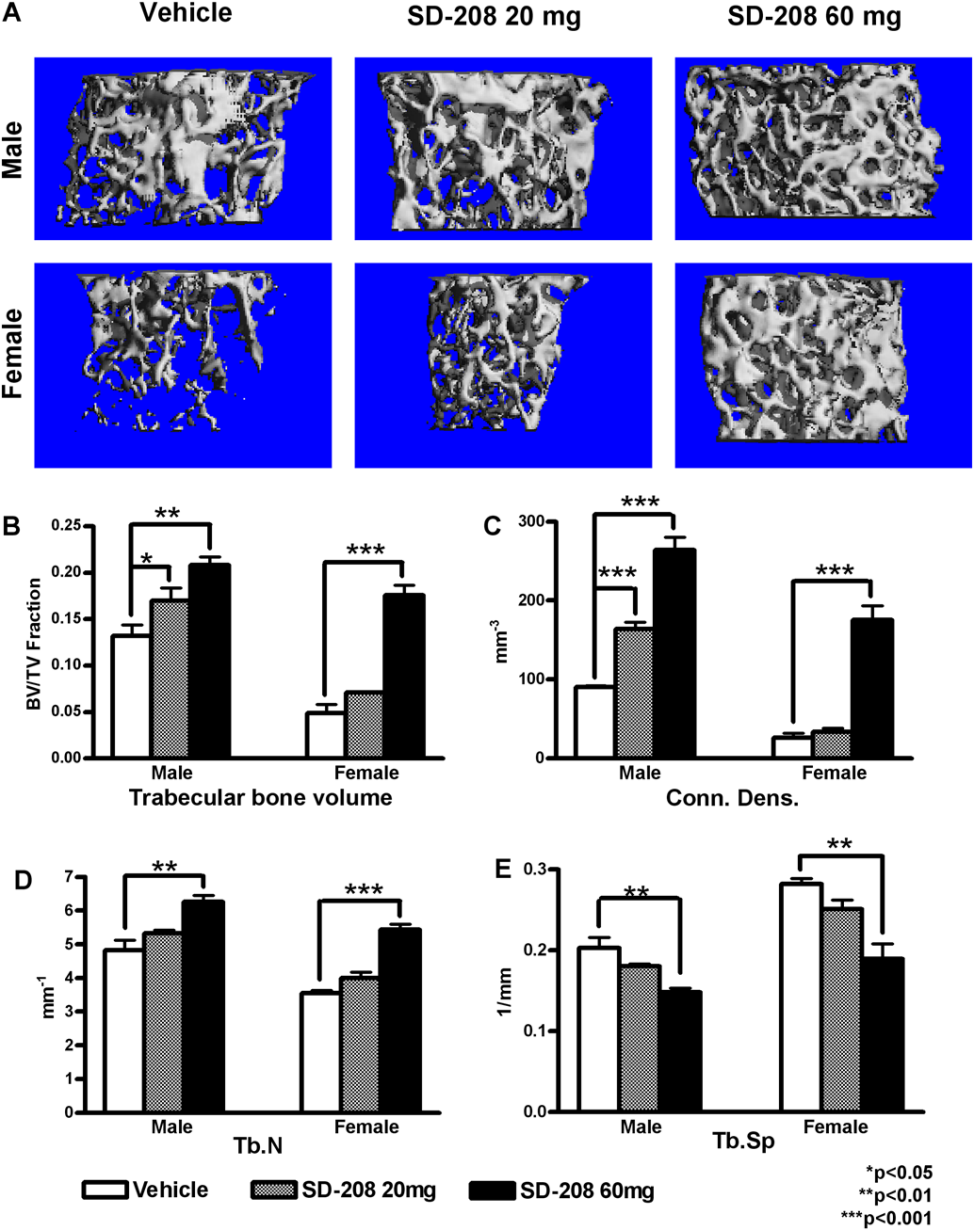
Pharmacologic TβRI inhibition increases trabecular bone volume. Micro-CT images show increased femoral trabecular bone volume following SD-208 treatment in male and female mice, relative to vehicle-treated controls (a). Quantitative analyses show that SD-208 increased trabecular bone volume (BV/TV, fraction) (b), connectivity density (c), and trabecular number (d), but decreased trabecular spacing (e) in male and female femora. Data represent mean±SEM (p<0.05, as determined by one-way ANOVA Newman-Keuls multiple comparison test).

To determine if the increased BMD resulted from changes in cortical or trabecular bone, dissected femora and tibiae were analyzed using micro-computed tomography (micro-CT). Reconstructed images of trabecular bone in the distal femur showed a dose-dependent increase in trabecular bone volume following 6 weeks of SD-208 treatment in both male and female mice (Fig. 3a). This increase in trabecular bone was noted in the secondary spongiosa and did not extend to the diaphysis (Figure S1). At the high dose, SD-208 increased the femoral trabecular bone volume of male and female mice by 57.6% and 264%, respectively (Fig. 3b, Table 1). Remarkably, high-dose SD-208 increased the trabecular density of male and female femora by 192% and 581%, respectively (Fig. 3c). Increases in trabecular number and thickness were associated with a corresponding decrease in trabecular separation following treatment with SD-208 (Fig. 3d, 3e, Table 1). As shown by these and other parameters, SD-208 greatly improved trabecular bone microarchitecture in male and female femora and tibiae (Table 1). In contrast, SD-208 caused no significant differences in measured cortical bone parameters (Table 2). Therefore, the effect of 6 weeks of pharmacologic inhibition of TβRI function on BMD appears to be specific to the trabecular bone.

**Table 1:** Trabecular bone structural parameters are affected by TβRI inhibition.

	Male						Female					
	Tibia			Femur			Tibia			Femur		
	Vehicle	20 mg SD-208	60 mg SD-208	Vehicle	20 mg SD-208	60 mg SD-208	Vehicle	20 mg SD-208	60 mg SD-208	Vehicle	20 mg SD-208	60 mg SD-208
**TBV**	0.11±0.006	0.182±0.018**	0.20±0.009**	0.132±0.017	0.169±0.013*	0.208±0.008**	0.09±0.009	0.113±0.007	0.161±0.014**	0.048±0.009	0.071±0.0009	0.175±0.011***
**DT.Tb.Th**	0.048±0.0006	0.049±0.002	0.047±0.0004	0.049±0.0012	0.049±0.003	0.046±0.0003	0.045±0.002	0.048±0.0009	0.046±0.0005	0.039±0.002	0.045±0.001*	0.048±0.0005*
**DT.Tb.N**	4.44±0.19	5.53±0.10***	6.19±0.15***	4.82±0.29	5.32±0.09	6.27±0.18**	3.34±0.15	3.69±0.15	5.22±0.39**	3.54±0.08	3.99±0.17	5.43±0.16***
**DT.Tb.Sp**	0.20±0.01	0.16±0.004	0.15±0.003*	0.20±0.01	0.18±0.002	0.14±0.004**	0.30±0.014	0.26±0.012	0.11±0.016***	0.28±0.006	0.25±0.011	0.18±0.018**
**Conn.Dens.**	60.52±4.13	129.6±12.06**	197.3±15.16***	90.32±1.61	163.1±8.68***	264.3±16.09***	47.13±5.21	50.55±6.01	138.2±25.01**	25.7±6.13	33.48±4.95	175.2±17.97***
**TRI SMI**	2.36±0.06	1.98±0.13	1.97±0.082	2.39±0.11	2.07±0.04*	1.78±0.009***	2.33±0.07	2.15±0.12	2.34±0.044	3.28±0.14	3.21±0.048	2.09±0.106***
**TRI DA**	2.13±0.06	2.11±0.07	1.92±0.06	1.33±0.009	1.42±0.04	1.33±0.03	2.34±0.066	2.6±0.023**	2.01±0.089***	1.4±0.106	1.4±0.006	1.42±0.006

Micro-computed tomography was used to assess several quantitative parameters of trabecular bone structure. The mean values and standard deviations are presented here. Significant differences between vehicle and SD-208 treated groups are indicated (*p<0.05, **p<0.01, ***p<0.001).

**Table 2:** Cortical bone structural parameters are not affected by TβRI inhibition.

	Male			Female		
	Vehicle	SD-208 20 mg	SD-208 60 mg	Vehicle	SD-208 20 mg	SD-208 60 mg
**Cort. CSA**	0.183±0.019	0.182±0.016	0.172±0.007	0.155±0.007	0.156±0.006	0.152±0.003
**Cort. Th.**	0.201±0.005	0.178±0.006	0.186±0.005	0.186±0.011	0.195±0.005	0.188±0.004
**Total CSA**	0.329±0.038	0.346±0.035	0.405±0.025	0.281±0.017	0.285±0.015	0.281±0.007
**Perios. Perim.**	1.057±0.098	1.202±0.081	0.986±0.057	0.964±0.047	0.977±0.045	0.958±0.017
**Diam. Mid Shaft**	0.592±0.017	0.546±0.017	0.577±0.004	0.554±0.012	0.555±0.005	0.560±0.010
**Med. Area**	0.182±0.040	0.218±0.098	0.214±0.019	0.105±0.009	0.109±0.008	0.110±0.005
**Endosteal Perim**	0.761±0.078	0.891±0.233	0.729±0.055	0.571±0.049	0.596±0.038	0.614±0.020
**Mid Diam**	0.447±0.054	0.427±0.077	0.551±0.013	0.352±0.008	0.351±0.003	0.343±0.011

Micro-computed tomography was used to assess several quantitative parameters of cortical bone structure. The mean values and standard deviations are presented here.

### Inhibition of TβRI affects both osteoblasts and osteoclasts

**Figure 4:**
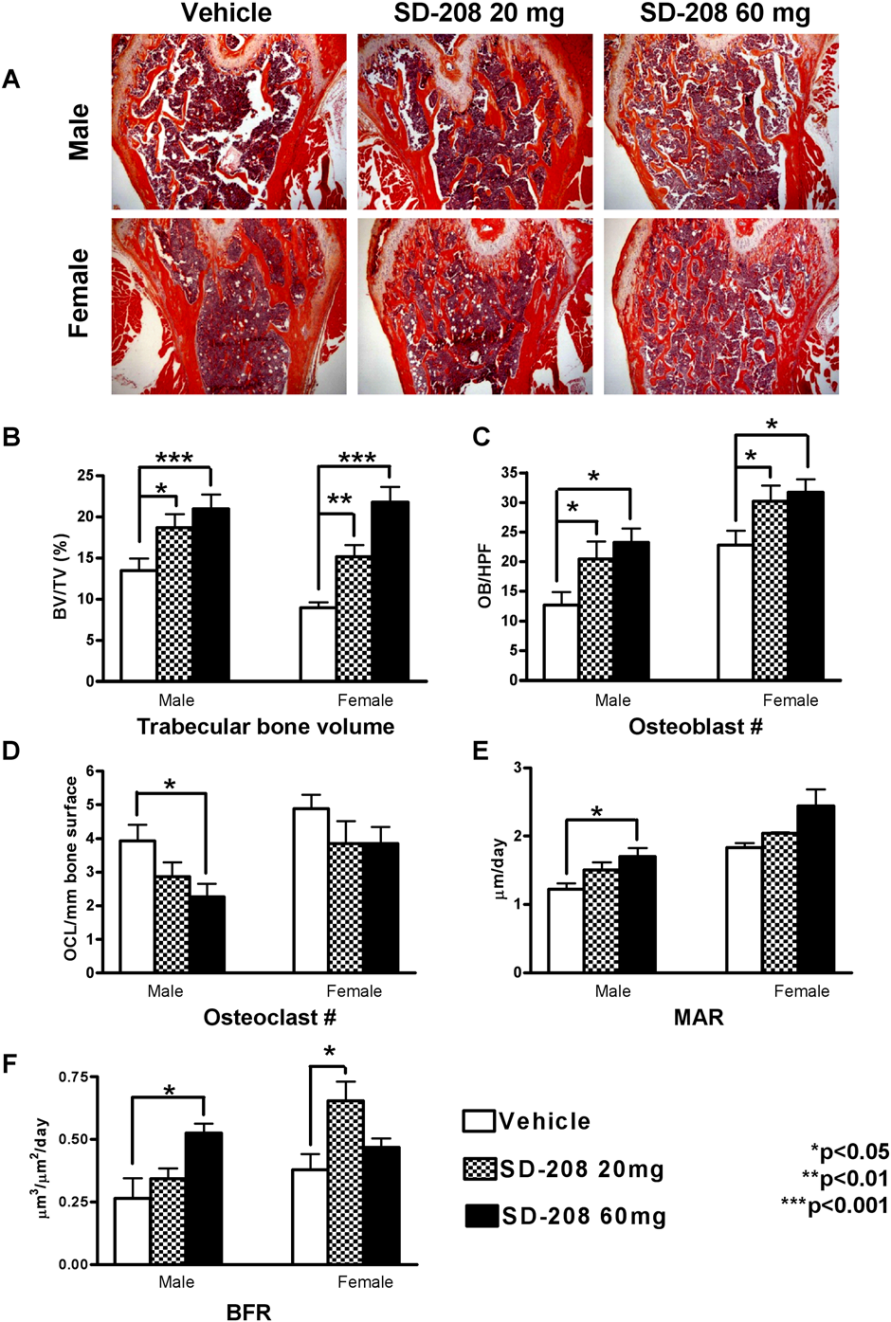
Pharmacologic TβRI inhibition increases osteoblast numbers but reduces osteoclast numbers. Representative H&E stained sections of femoral bone show the SD-208-dependent increase in trabecular bone in male and female mice (a). Histomorphometry shows that SD-208 increases trabecular bone volume in the femur (b) and tibia (data not shown), as well as osteoblast number (c) in a dose-dependent manner for male and female mice. Osteoclast numbers are reduced by SD-208 (60 mg/kg) in male mice (d). Dynamic histomorphometry of male mouse lumbar vertebrae shows that SD-208 treatment (60 mg/kg) increased mineral apposition rate (MAR) (e) and bone formation rate (BFR) (f). Data represent mean±SEM (*p<0.05, **p<0.01, ***p<0.001, as determined by one-way ANOVA Newman-Keuls multiple comparison test).

Increased BMD may be due to increased osteoblast activity, reduced osteoclast activity or both. Quantitative histomorphometry confirmed the SD-208 dose-dependent increase in trabecular bone that was observed by micro-CT (Fig. 4a). The significantly increased bone volume (Fig. 4b) was accompanied by a TβRI inhibitor dose-dependent increase in osteoblast number (Fig. 4c). Importantly, even the most specific low dose of SD-208 (20 mg/kg) caused significant increases in male and female bone volume and osteoblast numbers (p<0.05). In addition, the osteoclast numbers were reduced in the femora of SD-208 treated mice (Fig. 4d). Bones from male mice treated with the highest dose of SD-208 had twice as many osteoblasts and half as many osteoclasts as the vehicle-treated controls (Fig. 4).

These data suggest that inhibition of TGF-β signaling increases bone mass by enhancing bone formation and inhibiting bone resorption. Dynamic histomorphometry revealed that SD-208 stimulates a dose-dependent increase in the mineral apposition rate and bone formation rate in male mice (Fig. 4e, 4f). Female mice showed the same trend. Collectively, these analyses demonstrate that TβRI inhibitors increase bone mass in mature mice via anabolic and anti-catabolic mechanisms.

### TβRI inhibition promotes osteoblast differentiation and inhibits osteoclast differentiation

**Figure 5:**
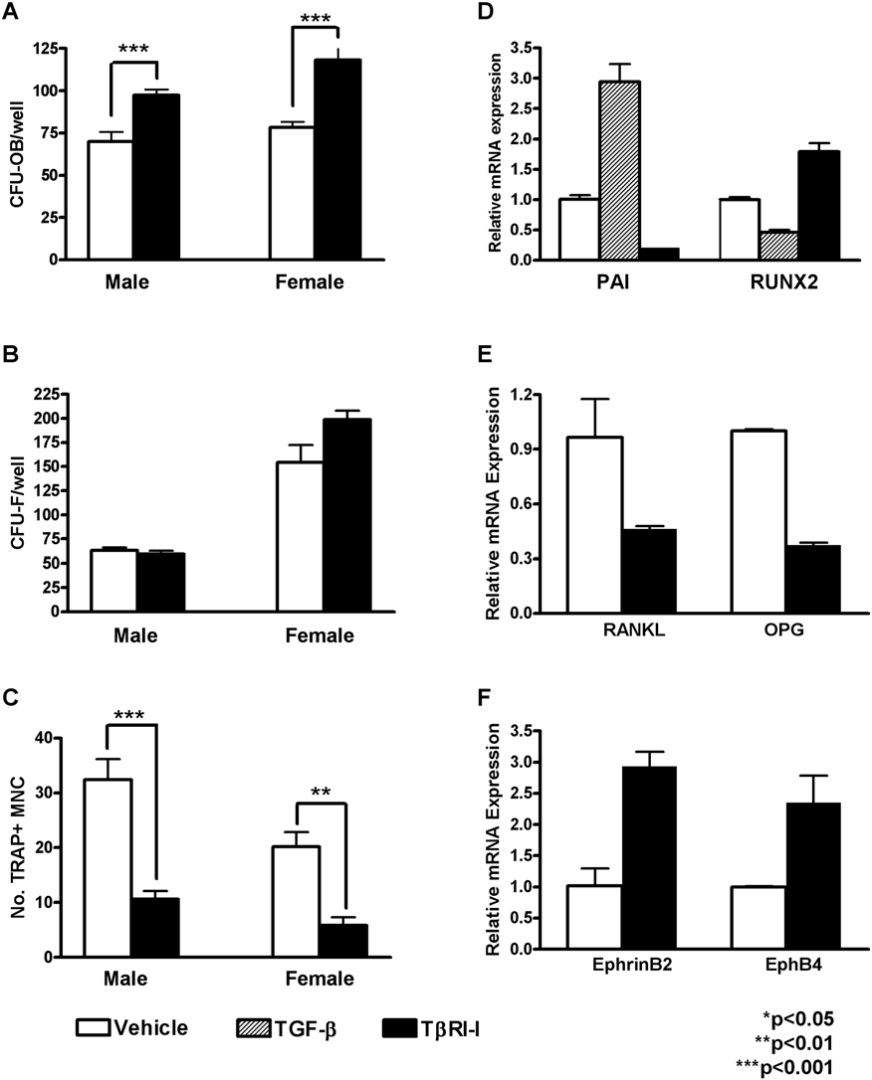
TβRI inhibition promotes osteoblast differentiation and bone deposition but inhibits osteoclast differentiation. Bone marrow isolated from male and female mice treated with SD-208 (60 mg/kg) has increased numbers of osteoblast colony forming units (CFU-OB) (a) with no change in the number of colony forming units (CFU-F) (b). The number of TRAP-positive multinucleated cells (TRAP+ MNC) is lower in cultures from SD-208 treated mice than from vehicle-treated controls (c). Primary calvarial osteoblasts treated with TβRI-inhibitor SB431542 (10µM) or vehicle for 48 h show altered mRNA expression of PAI-1 (d) and several osteoblast and osteoclast regulatory factors including Runx2 (d), RANKL and OPG (e), and ephrinB2 and EphB4 (f). Data represent mean±SEM (*p<0.05, **p<0.01, ***p<0.001, as determined by unpaired *t*-test).

To determine if the changes in osteoblast and osteoclast numbers and activity resulted from changes in cell differentiation, bone marrow stromal cells that were isolated from vehicle- and SD-208-treated mice were examined ex vivo in osteoblast or osteoclast differentiation assays (Fig. 5a–5c). In vivo exposure to SD-208 enhanced the osteoblast differentiation (CFU-Ob, Fig. 5a) with no detectable effect on osteoprogenitor recruitment (CFU-F, Fig. 5b). Conversely, marrow stromal cells from mice treated with SD-208 formed fewer multinucleated cells that express the functional osteoclast marker TRAP (Fig. 5c). Thus, in vivo inhibition of TβRI with SD-208 promotes osteoblast differentiation and inhibits osteoclast differentiation.

To investigate the effect of TβRI inhibitors on the expression of osteoblast and osteoclast regulatory factors, we utilized primary calvarial osteoblasts, which retain the capacity to differentiate into mineralizing osteoblasts, and have an intact autocrine TGF-β regulatory pathway. As in calvarial explants treated with SD-208 (Fig. 1c), another ATP-competitive TβRI kinase inhibitor, SB431542, inhibits the expression of the TGF-β-inducible gene, PAI-1 [30], in primary calvarial osteoblasts (Fig. 5d). As shown previously [22], Runx2 expression was reduced after 48 h of treatment with added TGF-β (Fig. 5d). In contrast, TβRI inhibitors induce Runx2 expression, consistent with the increased osteoblast numbers, bone formation rate, and osteoblast differentiation potential observed in SD-208-treated mice (Fig. 4c, 4f, 5a, 5d).

RANK ligand (RANKL) promotes osteoclast differentiation, function and survival [21]. After 48 h of treatment, TβRI inhibitors reduced the expression of RANKL mRNA by approximately 50% compared with the mRNA levels observed in vehicle-treated primary calvarial osteoblasts (Fig. 5e). The reduced expression of this osteoclastogenic factor is consistent with the decreased osteoclast numbers and differentiation capacity observed in SD-208-treated mice (Figs. 4d and 5c). However, RANKL function is antagonized by osteoprotegerin, the expression of which is also reduced by TβRI-I treatment. Similar results were observed after 2 h of TβRI-I treatment (data not shown). Though the inhibition of TGF-β signaling impacts both of these critical regulators of osteoclast differentiation and function, the relative RANKL/OPG ratio is unchanged. Therefore, the effect of inhibition TβRI function on other factors which regulate osteoblast and osteoclast function was investigated.

Recently, ephrin B2 and EphB4, a transmembrane ligand and receptor respectively, have been implicated as factors that couple osteoblast and osteoclast activities in bone metabolism [32]. Bidirectional signaling between ephrin B2, expressed by osteoblasts and osteoclasts, and EphB4 on osteoblasts increases osteoblast differentiation and inhibits osteoclast differentiation [32]. However, the ability of TGF-β to control ephrin signaling in bone metabolism has not been reported. Inhibition of TβRI function significantly increased the expression of both ephrin B2, the ephrin that inhibits osteoclast differentiation (Fig. 5f), and EphB4, the Eph receptor that induces osteoblast differentiation. TGF-β signaling crosstalk with the ephrin pathway may contribute to the anabolic and anti-catabolic effects of SD-208 on bone, though additional experiments are needed to establish a functional link. By affecting osteoblast and osteoclast differentiation, numbers and activity (Figs. 4c–f, 5a, 5c), the TβRI inhibitor dramatically shifts bone toward a state of metabolic anabolism.

### TβRI inhibitors increase bone matrix mineral concentration, material properties and fracture resistance

**Figure 6:**
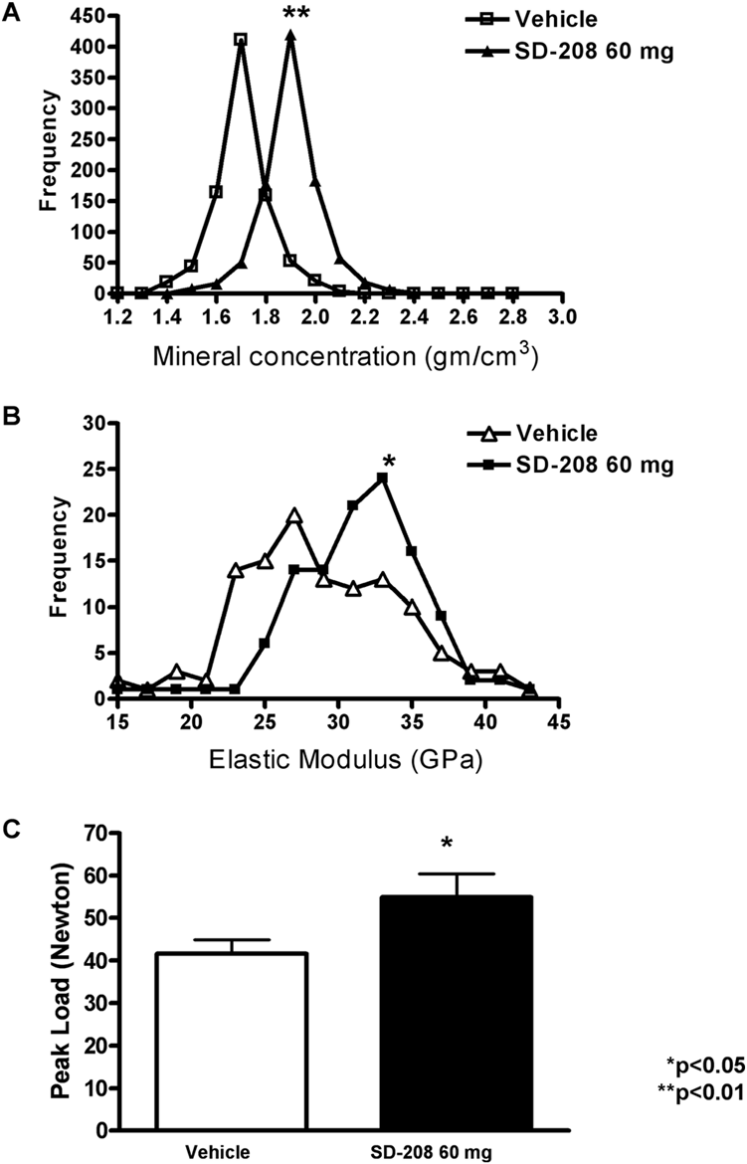
TβRI inhibitors increase bone mechanical and material properties. Analysis of each pixel from XTM scans of femora show that SD-208 (60 mg/kg) increases bone matrix mineral concentration with a mean of 1.90 g/cm^3^+/−0.066, relative to a mean mineral concentration of 1.54 g/cm^3^+/−0.069 for vehicle-treated controls (p<0.05, as determined by unpaired *t*-test) (a). Analysis of elastic modulus values from nanoindents applied to tibial cortical bone showed a similar shift (p<0.05) (b). Unconfined compression testing of vertebrae from male mice treated with vehicle or SD-208 (60 mg/kg) shows an increased peak load-to-failure following TβRI inhibition (p<0.05) (e).

The net effect of TβRI inhibitors on bone is increased BMD, which reflects both bone mass and mineral concentration (Fig. 2). With the monochromatic light from synchrotron radiation, X-ray tomographic microscopy (XTM) permits direct quantification of the mineral concentration of bone matrix with an 8 µm resolution [33]. Analyses of femoral bone showed that SD-208 treatment resulted in a higher degree of mineralization of bone matrix (Fig. 6a). The SD-208-dependent increase in mineral concentration was evident in both the diaphysis and epiphysis (data not shown), suggesting that the mineralization of both cortical and trabecular bone were affected.

Mineral concentration is a major determinant of bone matrix material properties [34]. Using nanoindentation, material properties such as the elastic modulus and hardness of bone matrix can be determined independently of changes in bone mass or structure [35], [36]. We have previously used this approach to demonstrate that TGF-β signaling in osteoblasts regulates the elastic modulus and hardness of bone matrix in genetically-modified mice [10]. Treatment of mice with SD-208 increased the elastic modulus of cortical bone relative to vehicle-treated controls. Although the measured modulus values in each group overlapped, more than half of the measurements in vehicle-treated bone were below 28 GPa, whereas less than a quarter of the values measured in SD-208-treated mice were in the same range (Fig. 6b).

Treatment with the TβRI inhibitor SD-208 affects bone on several levels, including bone mass (Figs. 2–4), bone mineral concentration and bone matrix material properties (Figs. 6a, 6b). These observations led us to perform macro-mechanical testing to evaluate the ability of SD-208-treated bones to resist fracture. Compression testing of vertebrae showed that inhibition of the TβRI kinase increased the load-to-failure relative to vehicle-treated controls (Fig. 6c, Table 3). When the femora were tested using notched or unnotched three-point bending, SD-208-dependent differences in peak load, stiffness, or fracture toughness were not observed (Table 4). The increased load-to-failure of SD-208-treated vertebral bone, but not femoral bone, is entirely consistent with the increase in trabecular but not cortical bone volume after 6 weeks of SD-208 treatment. Together these data demonstrate that TGF-β inhibitors drive functionally significant and coordinated increases in trabecular bone mass, mineral concentration and bone matrix material properties.

**Table 3:** Macromechanical testing of vertebrae.

	Male Vertebrae	
	Vehicle	SD-208 60 mg
**Peak Load**	41.55±3.34	54.95±5.45*
**Stiffness**	98.61±11.52	111.1±18.70

Mean values±SEM for macromechanical tests of vertebral peak load and stiffness are shown. The significance of differences between vehicle and SD-208 (60 mg/kg) treated groups is indicated with p values (*p<0.05).

**Table 4:** Macromechanical testing of femora.

	Male Femora	
	Vehicle	SD-208 60 mg
**Peak Load**	16.09±0.90	15.55±0.52
**Stiffness**	40.32±5.59	47.10±1.40
**Fracture Toughness**	4.367±0.783	4.755±0.509

Mean values±SEM for macromechanical tests of femoral peak load, stiffness and fracture toughness are shown.

## Discussion

Here we explored the role of TGF-β signaling in postnatal bone by systemic administration of a TβRI inhibitor to mature mice. Pharmacologic inhibition of TGF-β signaling resulted in dose-dependent increases in BMD, trabecular microarchitecture, bone matrix elastic modulus and mineral concentration. These coordinated changes in bone mass and parameters of bone quality improved the ability of vertebral bone to resist fracture. By targeting key regulatory pathways in osteoblasts and osteoclasts, TβRI inhibitors increased the number of osteoblasts and the bone formation rate, while reducing osteoclast numbers. Therefore, TβRI inhibition elicits both anabolic and anti-catabolic activities to improve bone quality.

The TβRI inhibitor-dependent increase in tibial BMD exceeded the physiologic increase in BMD over this time period or those induced by comparable regimens utilizing clinically available bisphosphonates or PTH [31], [37]. TβRI inhibitors may have more profound effects since they both stimulate bone formation and inhibit bone resorption, rather than the uncoupled effects of PTH to stimulate osteoblast activity or bisphosphonates to inhibit osteoclast activity. The effects of the TβRI inhibitor on adult bone are consistent with the developmental bone phenotypes of mice with partial inhibition of TGF-β/Smad signaling, as observed in Smad3+/− mice or DNTβRII mice that express a dominant negative TGF-β type II receptor in osteoblasts [7], [10]. In contrast, more complete inhibition of TGF-β signaling in Smad3−/− and TGF-β1−/− mice is associated with low bone mass and poor bone quality, which may result, in part, from the significant systemic effects of Smad3 and TGF-β1 deletion [5], [8], [9].

Some effects of TβRI inhibition on bone resulted from the reduction in osteoclast numbers and differentiation potential in SD-208-treated mice (Figs. 4d, 5c). This in vivo response is striking because TGF-β has been shown to inhibit and promote osteoclast differentiation in vitro, depending on the timing, dose and experimental cell population [12]. TGF-β can act by binding directly to its receptors on osteoclasts and their progenitors, or by acting on osteoblasts to regulate the expression of osteoclast regulatory factors, such as RANKL and OPG [6], [7], [23]–[25], [38]–[40]. Though the current study does not explore the extent to which SD-208 affects osteoclasts directly or indirectly through osteoblast-dependent mechanisms, SD-208 can directly inhibit osteoclast function in a purified osteoclast precursor population (Guise, personal communication). In addition, TβRI inhibitors regulated osteoblast expression of osteoclast regulatory factors such as RANKL, OPG, ephrin B2 and EphB4 (Fig. 5). Likely, a combination of direct and indirect mechanisms is responsible for the anti-catabolic and anabolic effects of TβRI inhibitors in vivo.

Treatment of mice with TβRI inhibitors resulted in increased osteoblast numbers and differentiation, and increased bone formation. Consistent with these data, reduced TGF-β signaling in Smad3+/− mice or DNTβRII mice also relieves the suppression of osteoblast differentiation by TGF-β, which is exerted by Smad3 and histone deacetylases [Bibr pone.0005275-Alliston2] [22,41], thereby contributing to increased BMD [Bibr pone.0005275-Filvaroff1] [7,10]. The increased osteogenic differentiation in response to TβRI inhibitors may also reflect a decrease in repression of BMP signaling by the inhibitory Smad6 [42]. Although changes in Smad6 expression were not observed in our experimental conditions, the BMP antagonist, Noggin, reversed some effects of TβRI inhibitors on gene expression (data not shown), affirming the previous observation that increased BMP signaling contributes to the osteogenic activity of TβRI inhibitors [42]. Therefore, despite the ability of TGF-β to promote or inhibit specific stages of osteoblast differentiation [13], the net effect of TβRI inhibitors on osteoblasts in vivo is to increase bone formation.

Ultimately, the ability of bone to resist fracture is the most clinically desirable outcome [11]. TβRI inhibition increased the peak load that vertebral bone can sustain prior to fracture, in part due to the potent anabolic effect of TβRI inhibitors on trabecular bone. Although a 6-week treatment with TβRI inhibitors was insufficient to increase cortical bone mass or geometry, it significantly increased the mineralization and material properties of cortical bone matrix, when measured using high-resolution XTM and nanoindentation. These data suggest that optimization of the dose or duration of therapy may result in detectable changes in cortical bone mass and macromechanical behavior. Furthermore, our data indicate that TGF-β signaling helps define bone matrix material properties postnatally as it does in development [10], although the effect was more modest than that observed in genetically modified mice. Although the elastic modulus of bone matrix often correlates with mineral content [1], Smad3 also regulates material properties independently of mineralization, as has recently been shown in skin [43]. The mechanisms by which TGF-β regulates the material properties of extracelullar matrices remain unknown.

In conclusion, pharmacologic inhibition of TGF-β signaling in postnatal bone increases bone quality. Coupling of osteoblast and osteoclast activity may be critical for the ability of TGF-β to coordinately control bone mass, architecture, and the material properties of bone. Therefore, therapies that produce a reliable reduction in TGF-β signaling may have significant clinical benefit in the treatment of diseases characterized by low bone mass and bone fragility. However, TβRI-inhibition may be counter-indicated for the treatment of existing bone fractures, where TGF-β plays a role in fracture repair. Additional studies evaluating the efficacy and potential sex-specificity of the mature skeletal response to TβRI inhibitors, particularly in ovariectomized animals, would be needed to determine their potential therapeutic value for post-menopausal osteoporosis. Careful consideration of safety is essential, given the critical role of TGF-β in normal physiological processes including the control of cell proliferation, differentiation, and apoptosis in many tissues.

## Materials and Methods

### Ethics Statement

In all studies, mice were handled and euthanized in accordance with approved institutional, national and international guidelines.

### TβRI inhibitor treatment

Four-week old male and female C57BL/6 mice were treated for 6 weeks with vehicle (1% methylcellulose) or SD-208 (20 mg/kg once daily or 60 mg/kg twice daily) by gavage. As described, SD-208 is a specific inhibitor of the TGF-β type I receptor, developed by Scios, Inc. [17]. Based on the mouse monitoring parameters of our treatment protocol, no adverse effects of SD-208 on mouse health were detected during the study. At 10 and 3 days prior to euthanasia, an intraperitoneal injection of calcein (Sigma C-0875, 0.02 mg/g) was administered to all mice. Forelimbs, hindlimbs, and spines were collected. For studies using SBE-luciferase mice [29], mice were treated with vehicle or 60 mg/kg SD-208 as above for 3 days, prior to an intraperitoneal injection of TGF-β1 (10 µg/kg). Five hours later, mice were administered luciferin (150 mg/kg) intraperitoneally, anaesthetized with isoflourane, and imaged 10 minutes later using a bioluminescence imaging system (Xenogen).

### Bone mineral density (BMD) measurement

BMD was measured using a PIXImus mouse densitometer (GE Lunar II, Faxitron Corp., Wheeling, IL) (N = 15/group). Total body measurement was performed excluding the calvarium, mandible and teeth. Regions of interest were defined as the distal femur and proximal tibia just beneath the growth plate (12×12 pixels) and the lower lumbar spine (20×50 pixels). Values were expressed as percentage change in BMD over the pretreatment scan.

### Histomorphometry

For demineralized bone histomorphometry, tissues were fixed for 48 h in 10% formalin, demineralized in 10% EDTA for 2 weeks, and embedded in paraffin to generate 3.5 µm longitudinal sections. Trabecular bone volume of the secondary spongiosa (BV/TV%) and osteoblast number (N.Ob/high power field) were measured on hematoxylin and eosin stained sections of the distal femur, proximal tibia, and lumbar vertebrae (N≥12 mice/group). Tartrate resistant acid phosphatase (TRAP) stained sections were used to quantify osteoclast number (N.Oc/BS/mm). Dynamic bone histomorphometry was performed on 7 µm thick sections of mineralized lumbar vertebrae embedded in methylmethacrylate using standard procedures. The mineral apposition rate (MAR, µm/day) and bone formation rate (BFR/BS, µm^3^/µm^2^/day) were measured on vertebral trabecular bone using fluorescence microscopy to visualize calcein labels as described [44].

### Micro-computed tomography (micro-CT)

Formalin fixed tibiae and femora were imaged with micro-CT using a microCT-40 (Scanco Medical AG, Bassersdorf, Switzerland) using a voxel size of 12 µm in all dimensions (N≥12 mice/group). The region of interest comprised 240 transverse CT slices representing the entire medullary volume with a border lying approximately 100 µm from the cortex [45]. Morphometric variables were computed using direct, three-dimensional techniques that do not rely on assumptions about the underlying structure. Fractional bone volume (BV/TV, Fraction) and architectural properties of trabecular reconstructions, apparent trabecular thickness (Tb.Th., µm), trabecular number (Tb.N., mm^−1^), trabecular spacing (Tb.Sp., 1/mm), and connectivity density (Conn.D., mm^−3^) were calculated as described [46].

### Cortical bone assessment by micro-CT

The CT images of the mid-diaphysis of the tibia were segmented into bone and marrow regions by applying a visually chosen, fixed threshold for all samples, after smoothing the image with a three-dimensional Gaussian low-pass filter. The outer contour of the bone was found automatically with the built-in Scanco iterative contouring tool. Total area (TA) was calculated by counting all voxels within the contoured bone area, (BA) by counting all voxels that were segmented as bone, and marrow area (MA) was calculated as TA-BA. This calculation was performed on all 30 slices (1 slice = 12.5 µm), using the average for the final calculation. The outer and inner perimeter of the cortical midshaft was determined by a three-dimensional triangulation of the bone surface (BS) of the 30 slices, and cortical parameters were calculated as described [47].

### Marrow stromal cell differentiation assays

Bone marrow stromal cells were flushed from 6 femora and tibiae per treatment group, collected by centrifugation (1500 rpm, 10 minutes), resuspended (αMEM, 10% FCS), and incubated for 2 h at 37°C. For osteoblast assays, cells were cultured in αMEM, 15% FBS, 50 µg/ml ascorbic acid, and 10 mM β-glycerophosphate. The number of alkaline phosphatase-positive osteoblast progenitor forming colonies (CFU-F) and Alizarin Red-positive osteoblast forming colonies (CFU-OB) was quantified microscopically after 9 or 28 days of culture, respectively, as described [Bibr pone.0005275-Giuliani1] [48,49]. For osteoclast progenitor assays, non-adherent cells were cultured for 6 days in 10% αMEM, 1% FBS and 10^−8^ M 1α,25(OH)_2_ vitamin D_3_. Cultures were fixed and stained for microscopic quantification of multinucleated (MNC) TRAP+ cells.

### Tissue culture, RNA isolation, and quantitative reverse transcription PCR

Calvarial explants were isolated from 10 day old SBE-Luc mice and cultured overnight in DMEM supplemented with 10% fetal bovine serum and 5 ng/ml TGF-β1 in the presence of either 150 nM SD-208 or an equivalent volume of vehicle (1% methylcellulose). Following culture, explants were moved to media containing luciferin (150 mg/ml) for immediate visualization of luciferase reporter activity with a bioluminescent imaging system (Xenogen). Explants were then crushed in liquid nitrogen using a mortar and pestle prior to additional tissue disruption in Trizol with a Omni-GLH homogenizer (Omni Scientific). Following Trizol extraction, RNA was further purified using RNeasy columns (Qiagen).

Primary calvarial osteoblasts were isolated from 3 to 5-day old mice and cultured in osteogenic conditions as described [22]. Cells were treated with a commercially available TGF-β receptor type I inhibitory compound suspended in DMSO (SB431542, Sigma) for 48 h. All other cells received an equivalent quantity of DMSO in the presence or absence of TGF-β (5 ng/ml). Total RNA was purified using RNAeasy columns (Qiagen) and reverse transcribed for the analysis of gene expression. Transcripts were amplified using primers sets for PAI-1 5′-AACCAATTTACTGAAAAACTGCACAA-3′ (forward) and 5′-TCCGGTGGAGACATAACAGATG-3′ (reverse), Runx2 5′-CCCAGCCACCTTTACCTACA-3′ (forward) and 5′-CAGCGTCAACACCATCATTC-3′ (reverse), OPG 5′-AGAGCAAACCTTCCAGCTGC-3′ (forward) and 5′-CTGCTCTGTGGTGAGGTTCG-3′ (reverse), RANKL 5′-CACCATCAGCTGAAGATAGT-3′ (forward) and 5′-CCAAGATCTCTAACATGACG-3′ (reverse), EphrinB2 5′-TCGAACTCCAAATTTCTACCC-3′ (forward) and 5′-TGCTTGGTCTTTATCAACCA-3′ (reverse), EphB4 5′-CAAAGTATGCAGAGCCTGTG-3′ (forward) and 5′-CCGGTAATACCCAATTCGAC-3′ (reverse). Results were detected based on amplicon binding of Sybr Green using quantitative RT-PCR and are representative of at least three independent experiments.

### X-ray tomography (XTM)

XTM studies were used to assess the degree of mineralization of the bone; procedures were based on the work of Kinney *et* al. [33]. Whole male mouse femora were scanned to determine the degree of bone mineralization (N = 3/group). Imaging was performed at the Advanced Light Source (ALS) on Beamline (8-3-2) at the Lawrence Berkeley National Laboratory by obtaining two-dimensional radiographs as the specimens were rotated through 180° in 0.5° increments. The radiographs were reconstructed into 2,500 slices by Fourier-filtered back projection with a 4.5 µm resolution. The attenuation coefficient (mm^−1^) of each pixel relates directly to bone mineral concentration. The degree of bone mineralization (DBM) was obtained from Eq. (1):
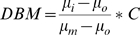
(1)where *μ_i_* is measured attenuation coefficient at pixel *i*, *μ_o_* is the attenuation coefficient of organic, *μ_m_* is attenuation coefficient of mineral, and *C* represents the density of hydroxyapatite.

### Nanoindentation

Dissected male mouse tibiae were embedded in a two-component epoxy resin (Stycast 1266) prior to sectioning with a precision low-speed saw to generate mid-tibial cortical bone surfaces for nanoindentation. A nanoindenter (Triboindenter, Hysitron, Minneapolis, MN) with a Berkovich tip was used to evaluate polished samples (0.25 µm) under dry conditions as described [10]. Indents were applied using a trapezoidal loading profile with a loading rate of 200 µN/second, peak load of 600 µN, and a hold period of 10 seconds. From the resulting load-deformation curves, local elastic modulus and hardness were calculated as described [50]. Three sets of 20 nanoindentation points were performed in a line with a 5 µm separation. Statistical analyses show the mean and standard error of the median elastic modulus values for each of 3 individual animals per group.

### Macroscopic mechanical testing

Whole bone strength and load to failure were determined by mechanical testing of vertebrae and intact tibiae for at least 12 mice per treatment group as previously described [45]. Thawed bones were hydrated in saline for 1 h before testing at room temperature using a MTS 858 Bionex Test Systems load frame (MTS Systems Corp, Eden Prairie, MN). Vertebral bodies (L4) were prepared with flat and parallel cranial and caudal ends by removing the soft cartilage to expose the bone, prior to compression testing at a rate of 3 mm/minute. Tibiae were tested in a three-point bending configuration with their anterior side down on two horizontal supports spaced 7 mm apart; the central loading point was displaced downward at 0.1 mm/second on the posterior surface of the diaphysis at the midpoint of the bone length. For all tests, load-displacement data were recorded at 100 Hz (TestWorks 4.0, MTS). Curves were analyzed to determine measures of whole-bone strength, primarily peak load and stiffness [47]. Load-to-failure was recorded as the load after a 2% drop from peak load.

Fracture toughness testing was performed on at least 10 isolated femora per condition. Thawed samples were notched using a razor blade followed by a micronotching technique. Notches were evaluated to ensure that they were through-wall but notched less than 1/3 of the bone diameter. Samples were tested in 37°C HBSS in a three-point bending configuration with a custom-made rig for the ELF 3200 mechanical testing machine (ELF3200, Bose, EnduraTEC, Minnetonka, MN), in general accordance with ASTM Standard E-399 and E-1820 [Bibr pone.0005275-E3991] [51,52] and as previously described [53]. Scanning electron microscopy was used to image fracture surfaces to measure the crack area and point of failure. The fracture toughness, *K*_c_, was calculated using a stress-intensity solution for circumferential through-wall flaw in cylinders [Bibr pone.0005275-Arany1] [43,54]. Macro-mechanical testing was performed on male and female femora, with no SD-208-dependent differences observed in either group.

## Supporting Information

Figure S1 The diaphysis is not filled by trabecular bone following SD-208 treatment. Although increased trabecular bone in femora from SD-208-treated mice (60 mg/kg) is evident in reconstructed micro-CT images, the trabecualr bone does not extend past the distal third of the femur. The scale bar is 1 mm.The diaphysis is not filled by trabecular bone following SD-208 treatment. Although increased trabecular bone in femora from SD-208-treated mice (60 mg/kg) is evident in reconstructed micro-CT images, the trabecualr bone does not extend past the distal third of the femur. The scale bar is 1 mm.
(1.00 MB TIF)
