# Supplementary figures and images for: Pharmacologic Inhibition of the TGF-β Type I Receptor Kinase Has Anabolic and Anti-Catabolic Effects on Bone
Source: PLoS One. 2009 Apr 16;4(4):e5275. doi: 10.1371/journal.pone.0005275 (PMC2666804; doi:10.1371/journal.pone.0005275)

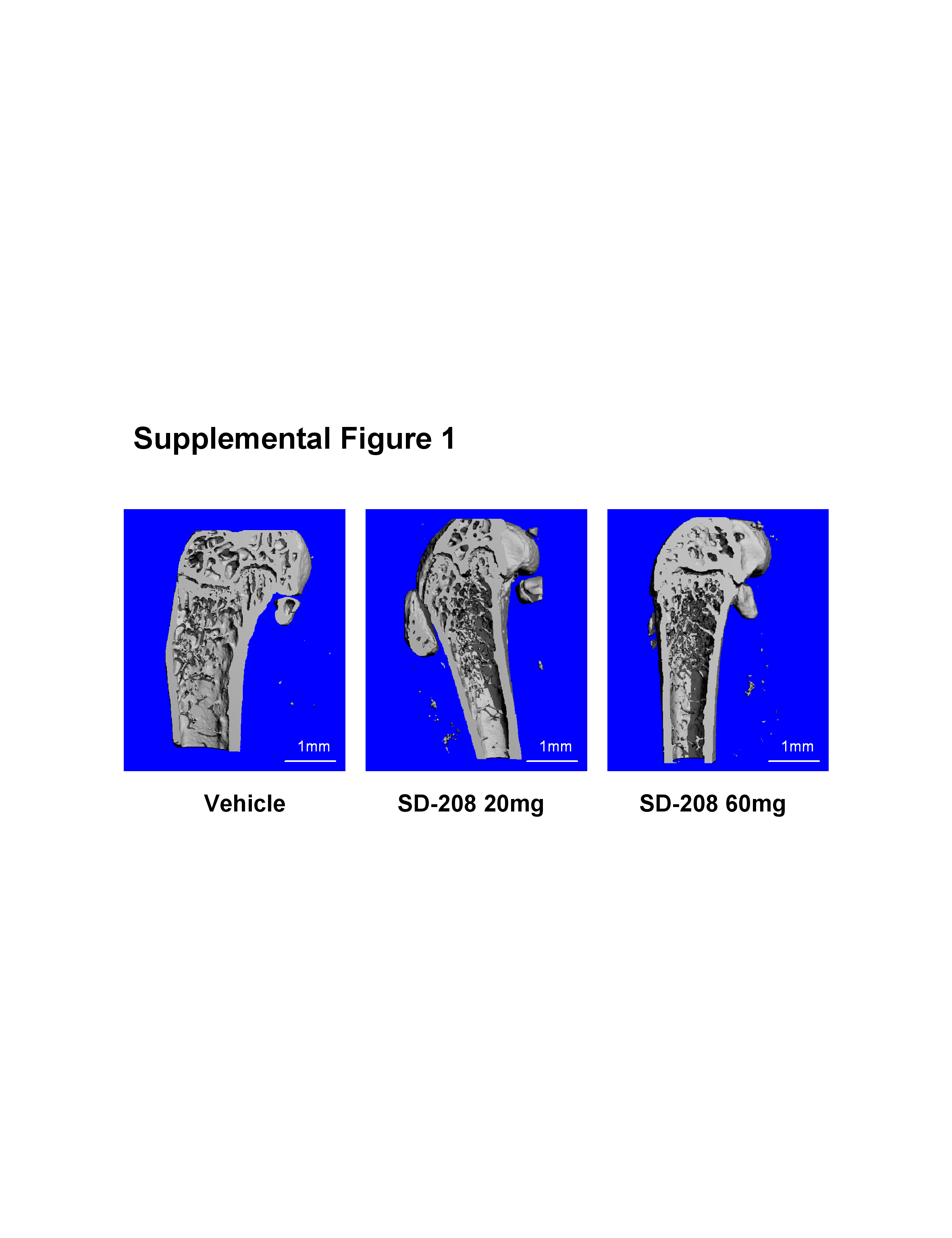

Supplement: Figure S1 — The diaphysis is not filled by trabecular bone following SD-208 treatment. Although increased trabecular bone in femora from SD-208-treated mice (60 mg/kg) is evident in reconstructed micro-CT images, the trabecualr bone does not extend past the distal third of the femur. The scale bar is 1 mm.The diaphysis is not filled by trabecular bone following SD-208 treatment. Although increased trabecular bone in femora from SD-208-treated mice (60 mg/kg) is evident in reconstructed micro-CT images, the trabecualr bone does not extend past the distal third of the femur. The scale bar is 1 mm. (1.00 MB TIF) [file pone.0005275.s001.tif]
